# Cardiovascular risk profile of patients with atherogenic dyslipidemia in middle age Lithuanian population

**DOI:** 10.1186/s12944-018-0851-0

**Published:** 2018-09-05

**Authors:** Sandra Kutkiene, Zaneta Petrulioniene, Aleksandras Laucevicius, Gabija Matuzeviciene, Vytautas Kasiulevicius, Emilija Petrulionyte, Justina Staigyte, Akvile Saulyte, Urte Gargalskaite, Egle Skiauteryte, Milda Kovaite, Egidija Rinkuniene

**Affiliations:** 1 Faculty of Medicine, Clinic of Cardiac and Vascular Diseases, Vilnius University, Vilnius, Lithuania; 2 Faculty of Medicine, Clinic of Internal Diseases, Family Medicine and Oncology, Vilnius University, Vilnius, Lithuania; 3 Faculty of Medicine, Vilnius University, Vilnius, Lithuania; 4 Vilnius University Hospital Santaros Klinikos, Vilnius, Lithuania

**Keywords:** Atherogenic dyslipidemia, Hypertriglyceridemia, Low-HDL cholesterol, Cardiovascular risk profile

## Abstract

**Background:**

Atherogenic dyslipidemia (AD) is a blood serum lipid profile abnormality characterized by elevation of triglycerides and reduced levels of high density lipoprotein cholesterol (HDL-C). It is associated with residual cardiovascular risk. This study evaluated and compared the risk profiles of patients with hypertriglyceridemia, low-HDL-C levels or AD, in order to understand, which lipid profile is associated with greater risk.

**Methods:**

During the period of 2009–2016 a population of 92,373 Lithuanian adults (men 40–54 years old and women 50–64 years old) without overt cardiovascular disease were analyzed. Data of 25,746 patients (68.6% women and 31.4% men) with hypertriglyceridemia and/or low HDL-C low levels were collected and used for further statistical analysis.

**Results:**

Participants with AD tend to have more unfavorable risk profile than participants with hypertriglyceridemia or low-HDL-C. AD tends to cluster with other atherogenic risk factors, such as arterial hypertension [odds ratio (OR) 1.96, 95% confidence intervals (CI) 1.87–2.01], smoking [OR 1.20, 95% CI 1.14–1.27], diabetes mellitus [OR 2.74, 95% CI 2.58–2.90], obesity [OR 2.92, 95% CI 2.78–3.10], metabolic syndrome [OR 22.27, 95% CI 20.69–23.97], unbalanced diet [OR 1,59, 95% CI 1.51–1.68], low physical activity [OR 1.80, 95% CI 1.71–1,89], CHD history in first degree relatives [OR 1.18, 95% CI 1.12–1.25] and total number of risk factors [OR 1.47, 95% CI 1.38–1.57].

**Conclusion:**

AD is associated with more unfavorable cardiovascular risk profile than hypertriglyceridemia or low-HDL cholesterol levels. Once identified AD should require additional medical attention since it is an important factor of residual cardiovascular risk.

## Background

Atherogenic dyslipidemia (AD) is a lipid abnormality defined as the presence of elevated triglycerides (TG) and low high-density lipoprotein cholesterol (HDL-C). It is known to increase the risk of coronary events in patients with or without CAD [1, 2] as well as silent myocardial infarction and silent CAD in high-risk patients with type 2 diabetes [3]. Additional findings such as elevation of small dense low-density lipoprotein (sd-LDL) particles, elevated levels of apolipoprotein B (Apo-B), detection of large TG rich very low-density lipoproteins and oxidized LDL, as well as decreased number of small HDL particles contributes to AD vascular risk [4]. AD is a common lipid profile in patients with obesity, metabolic syndrome, insulin resistance, type 2 diabetes mellitus and coronary artery disease (CAD) [5–8]. It is not clear whether AD is associated with higher risk than hypertriglyceridemia or low-HDL alone. The aim of our study was to evaluate prevalence of AD, hypertriglyceridemia and low-HDL-C levels in middle-aged Lithuanian population and to compare cardiovascular risk profile of patients with these different lipid panel abnormalities, in order to understand which lipid profile is associated with higher prevalence of cardiovascular risk factors.

## Methods

Our study is a part of the ongoing LitHiR Primary Prevention Program. It was launched in Lithuania in 2006, in reference to unfavorable situation of cardiovascular morbidity and mortality in Lithuania, with an approval of the Local Research Ethics Committee. LitHiR program includes 40–54 years old men and 50–64 years old women without overt cardiovascular disease (CVD) from all regions of Lithuania. 94.8% (398/420) of all the primary care institutions participate in the project. During the period of 2009–2016 our study included 92,373 participants. Data of 25,746 patients with hypertriglyceridemia > 1.7 mmol/l and/or low-HDL-C (< 1.2 mmol/l for women and < 1 mmol/l for men) were collected and used for further statistical analysis. Serum total cholesterol (TC), calculated LDL-C, HDL-C, TG levels, non-HDL cholesterol, TG/HDL ratio, log TG/HDL index and plasma fasting glucose concentration were estimated. Patients’ blood samples were processed at the standardized laboratories in the participating centres. Cardiovascular risk profile of each participant was obtained. A detailed description of the LitHir program protocol is presented in Laucevicius et al. paper [9].

The overall cardiovascular risk was evaluated according to the SCORE (Systematic Coronary Risk Evaluation) risk estimation system. Metabolic syndrome (MS) was assessed by the National Cholesterol Education Program ATP III modified criteria.

Participants were divided into three groups according to the components of AD: first group included adults with AD, which was considered if TG were > 1.7 mmol/l and HDL-C was < 1.2 mmol/l in women and < 1.0 mmol/l in men. The second group was composed of people with hypertriglyceridemia, which was considered if TG were > 1.7 mmol/l and HDL-C were > 1.2 mmol/l in women and > 1.0 mmol/l in men. The third group included men and women with low HDL-C, which was considered if HDL-C was < 1.2 mmol/l in women and < 1.0 mmol/l in men and TG were < 1.7 mmol/l. LDL-C levels did not impact division into groups.

Women with AD were divided into 50–54 (*n* = 1560), 55–59 (*n* = 1252) and 60–64 (*n* = 1268) year groups. Men were divided into 40–44 (*n* = 1264), 45–49 (*n* = 1043) and 50–54 (*n* = 1102) year old groups and their cardiovascular risk profile was assessed.

### Statistics

Categorical variables were described through absolute frequencies and percentage, and continuous variables through mean and standard deviation (SD). Continuous variables were compared using T-test or the Mann-Whitney test. Categorical variables were compared by carrying out the chi-square test. Multivariate logistic regression was performed to assess factors associated with AD, hypertriglyceridemia and low-HDL-C. Variables considered in the multivariate analysis included low physical activity, unbalanced diet, smoking, arterial hypertension (AH), diabetes mellitus (DM), obesity, CHD history in a first-degree relatives, MS and having more than 3 risk factors. All reported *p*-values are two-tailed. A value of *p* < 0.05 was considered significant. The SPSS version 22 (SPSS Inc., Chicago, Illinois, USA) statistical package was used for all statistical calculations.

## Results

### Sample characteristics

During the period of 2009–2016 our study included 92,373 participants without overt cardiovascular disease. The average age of subjects was 52.15 ± 6.21 years. The sample consisted of 58.4% (*n* = 53,961) women and 41.6% (*n* = 38,412) men. Any type of dyslipidemia was diagnosed in 89.7% (*n* = 82,893) of the subjects.

### Characteristics of subjects with different types of dyslipidemia

During this study, subjects were divided into three groups according to their lipid panel. In overall LitHiR population 25,746 participants had one of the following lipid abnormalities: AD– 29.1% (*n* = 7489, 54.5% of them were women and 45.5% men), hypertriglyceridemia 80.0% (*n* = 20,593, 50.7% women and 49.3% men) or low-HDL-C levels 20.0% (*n* = 5153, 60.8% women and 39.2% men). The prevalence of AD in LitHiR population (*n* = 92,373) was 8.1%, hypertriglyceridemia - 22.3% and low-HDL-C levels - 5.6%.

### Cardiovascular risk profile of subjects with different types of dyslipidemia

Demographic, anthropometric and laboratory characteristics of participants with AD, hypertriglyceridemia and low-HDL-C levels groups are shown in Table 1. Participants in low-HDL-C group were statistically significantly older (52.41 ± 6.33 years) in comparison with other groups. Participants with AD tended to have higher prevalence of AH (69.0%), DM (22.6%), abdominal obesity (67.6%), MS (88.9%), unbalanced diet (71.0%), low physical activity (64.2%) and percentage of people having more than 3 risk factors (86.1%). In addition, prevalence of smoking (26.1%) and CHD in first degree relatives (29.1%) was higher in AD group than in low-HDL-C group. Participants in low-HDL group had more favorable risk profile: lower prevalence of DM (12.5%), AH (56.9%), MS (52.7%), smoking (22.3%), unbalanced diet (62.2%), low physical activity (56.5%). Participants in hypertriglyceridemia group had higher values of total cholesterol (6.74 ± 1.24 mmol/l), LDL-C (4.28 ± 1.16 mmol/l), HDL-C (1.45 ± 0.34 mmol/l), non-HDL cholesterol (5.29 ± 1.22 mmol/l) and SCORE index (2.27 ± 1.94) in comparison with other groups.

**Table 1:** Demographic, anthropometric and laboratory characteristics of participants

	Atherogenic dyslipidemia		Hypertriglyceridemia		Low-HDL-C		*p* value	*p* (AD vs. high-TG)	*p* (AD vs. low-HDL-C)	*p* (high-TG vs. low-HDL-C)
	*n* = 7489		*n* = 20,593		*n* = 5153					
	Mean	SD	Mean	SD	Mean	SD				
Age (years)	52.03	6.6	51.71	6.44	52.41	6.33	< 0.001	0.001	0.004	< 0.001
BMI (kg/m^2^)	31.91	5.63	30.21	5.30	29.99	5.95	< 0.001	< 0.001	< 0.001	0.027
SBP (mmHg)	137.61	16.95	136.85	16.79	133.87	16.36	< 0.001	0.002	< 0.001	< 0.001
DBP (mmHg)	84.93	9.71	84.66	9.73	82.87	9.31	< 0.001	0.093	< 0.001	< 0.001
HR (beats/min)	73.02	8.70	72.67	8.77	72.52	8.88	0.002	0.009	0.005	0.519
Glucose concentration (mmol/l)	6.06	1.90	5.76	1.47	5.55	1.27	< 0.001	< 0.001	< 0.001	< 0.001
Total cholesterol (mmol/l)	6.16	1.25	6.74	1.24	5.10	1.05	< 0.001	< 0.001	< 0.001	< 0.001
LDL-cholesterol (mmol/l)	3.89	1.12	4.28	1.16	3.53	0.97	< 0.001	< 0.001	< 0.001	< 0.001
HDL-cholesterol (mmol/l)	0.95	0.15	1.45	0.34	0.98	0.15	< 0.001	< 0.001	< 0.001	< 0.001
TG (mmol/l)	3.16	2.09	2.55	1.22	1.24	0.29	< 0.001	< 0.001	< 0.001	< 0.001
Non-HDL (mmol/l)	5.20	1.23	5.29	1.22	4.11	1.02	< 0.001	< 0.001	< 0.001	< 0.001
TG/HDL ratio	3.58	4.11	1.86	1.04	1.32	0.55	< 0.001	< 0.001	< 0.001	< 0.001
SCORE index	2.19	1.95	2.27	1.94	1.63	1.45	< 0.001	0.008	< 0.001	< 0.001
Atherogenic index (log (TG/HDL-C))	0.48	0.22	0.23	0.17	0.10	0.14	< 0.001	< 0.001	< 0.001	< 0.001
*Frequency*	n	%	n	%	n	%	*p*	*p*	*p*	*p*
*DM (%)*	1696	22.6%	3258	15.8%	644	12.5%	< 0.001	< 0.001	< 0.001	< 0.001
AH (%)	5170	69.0%	13,373	64.9%	2934	56.9%	< 0.001	< 0.001	< 0.001	< 0.001
Abdominal obesity (%)	5062	67.6%	11,485	55.8%	2764	53.6%	< 0.001	< 0.001	< 0.001	0.018
Smoking (%)	1953	26.1%	5319	25.8%	1148	22.3%	< 0.001	1	< 0.001	< 0.001
MS (%)	6657	88.9%	11,809	57.3%	2714	52.7%	< 0.001	< 0.001	< 0.001	< 0.001
3 or more CVD risk factors (%)	6448	86.1%	15,051	73.1%	3473	67.4%	< 0.001	< 0.001	< 0.001	< 0.001
CHD history in first degree relatives (%)	2176	29.1%	5716	27.8%	1311	25.4%	< 0.001	0.1	< 0.001	0.003
Unbalanced diet (%)	5319	71.0%	14,086	68.4%	3204	62.2%	< 0.001	< 0.001	< 0.001	< 0.001
Low physical activity (%)	4811	64.2%	11,737	57.0%	2910	56.5%	< 0.001	< 0.001	< 0.001	1
BMI < 25 (kg/m^2^) (%)	591	7.9%	2996	14.5%	1001	19.4%	< 0.001	< 0.001	< 0.001	< 0.001
BMI 25–30 (kg/m^2^) (%)	2451	32.7%	7950	38.6%	1877	36.4%	< 0.001	< 0.001	< 0.001	< 0.001
BMI 25–30 (kg/m^2^) (%)	2451	32.7%	7950	38.6%	1877	36.4%	< 0.001	< 0.001	< 0.001	< 0.001
BMI 25–30 (kg/m^2^) (%)	2451	32.7%	7950	38.6%	1877	36.4%	< 0.001	< 0.001	< 0.001	< 0.001

Abbreviations: *AD* atherogenic dyslipidemia, *high-TG* hypertriglyceridemia, *low-HDL-C* low high-density lipoprotein cholesterol, *BMI* body mass index, *SBP* systolic blood pressure, *DBP* diastolic blood pressure, *HR* heart rate, *TG* triglycerides, *non-HDL* non high-density cholesterol, *DM* diabetes mellitus, *AH* arterial hypertension, *MS* metabolic syndrome, *CVD* cardiovascular disease, *CHD* coronary heart disease, *BMI* body mass index

### Characteristics of men and women with AD according to age

**Fig. 1:**
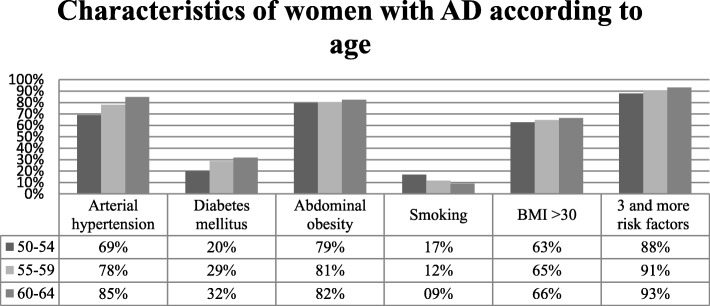
Characteristics of women with AD according to age

**Fig. 2:**
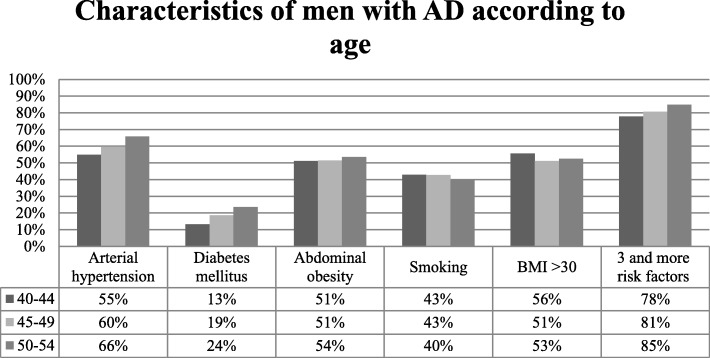
Characteristics of men with AD according to age

Analysis of participants with AD according to gender and age revealed that older women (60–64 years) with AD tend to have higher waist circumference (101.10 ± 13.23 cm), systolic blood pressure (141.56 ± 17.52 mmHg), TC (6.42 ± 1.26 mmol/l), LDL-C (4.19 ± 1.13 mmol/l), non-HDL-C (5.38 ± 1.25 mmol/l), serum glucose concentration (6.31 ± 2.10 mmol/l) and SCORE index (3.92 ± 2.35), as well as higher prevalence of low physical activity (70.7%), higher prevalence of AH (84.8%), DM (31.8%) and MS (96.0%) and higher percentage of people having more than 3 risk factors (93.1%). Older men (50–54 years) with AD tend to have lower TG (3.33 ± 2.11 mmol/l), higher blood pressure (systolic blood pressure - 137.78 ± 16.68 mmHg, diastolic blood pressure 85.81 ± 9.91 mmHg), serum glucose concentration (6.20 ± 2.16 mmol/l), SCORE index (3.40 ± 2.16), and percentage of people having more than 3 risk factors (84.9%), as well as higher prevalence of AH (65.9%), DM (23.5%) and MS (86.7%), and lower prevalence of CHD history in first degree relatives (24.4%) (Figs. 1 and 2).

### Logistic regression of risk factors for different types of dyslipidemia

According to LitHiR database, all analyzed risk factors, including main risk factors such as DM (OR: 2.74, 95% CI: 2.58–2.90), AH (OR: 1.96, 95% CI 1.87–2.01), obesity (OR: 2.92, 95% CI: 2.78–3.10) and smoking (OR: 2.74, 95% CI: 2.58–2.90) were significantly associated with AD and hypertriglyceridemia according to logistic regression with univariate models (Table 2). There was no association between isolated low HDL-C levels and smoking, CAD history in the first degree relatives and unbalanced diet.

**Table 2:** Risk factors for different types of dyslipidemia

Criteria		Atherogenic dyslipidemia		Hypertriglyceridemia		Low-HDL-C	
		OR (95% CI)	*p* value	OR (95% CI)	*p* value	OR (95% CI)	*p* value
Diabetes mellitus (%)	All	2.74 (2.58–2.90)	< 0.001	1.84 (1.76–1.93)	< 0.001	1.20 (1.11–1.31)	0.001
	Men	2.13 (1.94–2.34)	< 0.001	1.69 (1.57–1.81)	< 0.001	1.08 (0.94–1.25)	0.293
	Women	3.29 (3.05–3.55)	< 0.001	2.02 (1.90–2.15)	< 0.001	1.28 (1.15–1.42)	0.001
AH (%)	All	1.96 (1.87–2.01)	< 0.001	1.75 (1.69–1.80)	< 0.001	1.11 (1.05–1.18)	0.001
	Men	1.70 (1.59–1.83)	< 0.001	1.78 (1.70–1.87)	< 0.001	0.89 (0.82–0.98)	0.014
	Women	2.40 (2.23–2.59)	< 0.001	1.92 (1.84–2.02)	< 0.001	1.27 (1.18–1.37)	0.001
Smoking (%)	All	1.20 (1.14–1.27)	< 0.001	1.22 (1.18–1.27)	< 0.001	0.96 (0.90–1.03)	0.225
	Men	1.07 (0.99–1.14)	0.081	0.95 (0.91–0.99)	0.043	1.04 (0.95–1.14)	0.378
	Women	1.29 (1.17–1.42)	< 0.001	1.27 (1.19–1.36)	< 0.001	0.94 (0.83–1.06)	0.296
Obesity (%)	All	2.92 (2.78–3.10)	< 0.001	1.87 (1.81–1.93)	< 0.001	1.52 (1.44–1.61)	0.001
	Men	3.09 (2.88–3.32)	< 0.001	2.19 (2.09–2.30)	< 0.001	1.34 (1.22–1.47)	0.001
	Women	3.75 (3.46–4.06)	< 0.001	2.29 (2.19–2.40)	< 0.001	1.67 (1.54–1.80)	0.001
MS (%)	All	22.27 (20.69–23.97)	< 0.001	4.24 (4.10–4.38)	< 0.001	2.57 (2.43–2.72)	0.001
	Men	17.51 (15.97–19.19)	< 0.001	3.97 (3.78–4.17)	< 0.001	1.72 (1.57–1.89)	0.001
	Women	38.52 (33.68–44.04)	< 0.001	5.23 (5.00–5.48)	< 0.001	3.31 (3.07–3.57)	0.001
CHD history in first degree relatives (%)	All	1.18 (1.12–1.25)	< 0.001	1.12 (1.08–1.16)	< 0.001	0.97 (0.90–1.03)	0.340
	Men	1.30 (1.20–1.40)	< 0.001	1.20 (1.14–1.26)	< 0.001	0.96 (0.86–1.07)	0.427
	Women	1.13 (1.05–1.21)	< 0.001	1.11 (1.06–1.16)	< 0.001	0.97 (0.89–1.05)	0.414
Unbalanced diet (%)	All	1.59 (1.51–1.68)	< 0.001	1.47 (1.43–1.52)	< 0.001	1.03 (0.97–1.09)	0.297
	Men	1.47 (1.36–1.58)	< 0.001	1.49 (1.42–1.56)	< 0.001	0.96 (0.88–1.06)	0.407
	Women	1.68 (1.56–1.80)	< 0.001	1.42 (1.36–1.49)	< 0.001	1.09 (1.01–1.17)	0.029
Low physical activity (%)	All	1.80 (1.71–1.89)	< 0.001	1.35 (1.31–1.40)	< 0.001	1.25 (1.19–1.33)	< 0.001
	Men	1.79 (1.67–1.92)	< 0.001	1.42 (1.35–1.48)	< 0.001	1.13 (1.04–1.24)	0.007
	Women	1.87 (1.74–1.99)	< 0.001	1.40 (1.34–1.46)	< 0.001	1.33 (1.23–1.43)	0.001
3 or more CVD risk factors (%)	All	1.47 (1.38–1.57)	< 0.001	1.58 (1.51–1.64)	< 0.001	0.90 (0.84–0.96)	0.002
	Men	1.99 (1.76–2.25)	< 0.001	1.56 (1.46–1.67)	< 0.001	1.19 (1.04–1.36)	0.011
	Women	1.26 (1.16–1.36)	< 0.001	1.45 (1.37–1.52)	< 0.001	0.82 (0.76–0.89)	0.001

Abbreviations: *Low-HDL-C* low high density lipoprotein cholesterol, *AH* arterial hypertension, *MS* metabolic syndrome, *CHD* coronary heart disease, *CVD* cardiovascular disease

Further statistical analysis according to gender revealed that arterial hypertension, diabetes mellitus, obesity, metabolic syndrome, unbalanced diet and CHD history in first degree relatives were significantly associated with AD and isolated hypertriglyceridemia in both men and women. Unbalanced diet was significantly associated with low-HDL in men, however smoking did not show significant association with AD and low-HDL in men. There was significant association between unbalanced diet and low-HDL in women [OR: 1.09, 95% CI 1.01–1.17].

## Discussion

The prevalence of AD (8.1%) was similar to expected values, since we analyzed middle aged population without overt cardiovascular disease. Other authors reveal that the prevalence of AD varies from, approximately, 5.7% in working population [10], 9.9% in primary prevention patients with at least one cardiovascular risk factor [11], 27.1% in primary prevention patients of moderate to very high risk [12] and up to even 40.0% in patients who underwent coronary angiography due to myocardial infarction or unstable angina [2].

Our study also shows that participants with isolated hypertriglyceridemia had higher SCORE index than participants with AD. However participants with AD seemed to have worse risk profile. It is important to note, that using SCORE index might underestimate patients cardiovascular risk in patients with AD.

Low-HDL-C and high-TG were associated with low physical activity. It can be explained by association with obesity and increased caloric intake which leads to overproduction of triglyceride-rich lipoproteins (higher serum TG), increased expression of hepatic lipase and lower HDL-C [13]. This underlines a huge importance of lifestyle intervention (dietary restriction and increased exercise) in treating atherogenic dyslipidemia [14]. It has been reported that 5–10% weight loss can lower LDL-C by approximately 15% and TG approximately 20–30% and increase HDL-C approximately 8–10% [15]. According to our data women would benefit from weight loss the most, since unbalanced diet was strongly associated with AD in women (OR 1.68, 95% CI 1.56–1.80).

The prevalence of hypertriglyceridemia (22.3%) was similar to expected values. For example prevalence of hypertriglyceridemia in primary prevention patients with at least one cardiovascular risk factor in EURICA study was 20.8% [11].

The role of serum triglycerides concentration as an independent risk factor for cardiovascular disease remains controversial: some studies claim that hypertriglyceridemia is an independent risk factor, some studies reveals no significant association with CHD. For example, 29 prospective studies, which included 262,525 participants revealed moderate and highly significant associations between TG levels and CHD, however the impact of TG decreased after adjusting other factors association substantially attenuated [16]. On the other hand, meta-analysis of 68 long-term prospective studies including 302,430 people revealed no significant association between TG and CVD [17]. However, the cross-sectional nature of our study did not allow us to identify causal relationship.

The pattern of TG concentration changes in men according to age were similar to ones reported in literature. It is known that TG concentration in men tends to increase progressively with age reaching peak values at the age of 40 to 50 years, and shows a slight decline thereafter. TG concentration in women increase throughout their lifetime [18, 19]. In our study men had highest TG at the age of 40–44 years and TG concentration was lower in older men. The elevation of TG concentration in women was similar in all age groups.

Despite the fact, that the prevalence of dyslipidemia in Lithuania is high (89.7%), the prevalence of low-HDL-C (5.6%) was unexpectedly low, since reported prevalence of low-HDL in primary prevention patients with at least one cardiovascular risk factors is 22.1% [11] and even 56.5% in PERU MIGRANT study [20].

Low-HDL concentration is an independent risk factor of CHD irrespective of sex, race and ethnicity [21]. It is inversely associated with weight, abdominal circumference, TG concentration, number of small dense LDL particles, systemic inflammatory response and smoking [22]. However, the evidence supporting cardio protective role of high HDL-C levels are lacking, since drugs with effect of increasing HDL-C (fibrates, CETP inhibitors and nicotine acid) have failed to show benefit in decreasing incidence of coronary events and mortality rates in patients on statin therapy [23–25]. Participants in low-HDL group showed more favorable risk profile than other groups, which contributes to the notion that the quality of HDL particles plays an important role in the pathogenesis of CHD and further studies are needed. Walter reported that plasma HDL-C levels decrease in males during adolescence and early adulthood, but in elder age they are unchanged or even slightly increased [26]. In contrast women’s HDL-C levels remains stable throughout their lifetime, however, menopause often causes a slight decrease in HDL-C concentration [27]. In our study the differences of HDL-C levels in different age groups of both men and women were statistically insignificant.

The cross-sectional nature of our study did not allow us to identify causal relationship.

## Conclusion

Hypertriglyceridemia and low-HDL-C play an important role in residual CV risk profile. AD was associated with more unfavorable risk profile than hypertriglyceridemia or low-HDL cholesterol. AD was reported almost in one of ten adults without overt cardiovascular disease in Lithuania. Once identified AD should require additional medical attention.
